# Synergistic strengthening effect of nanocrystalline copper reinforced with carbon nanotubes

**DOI:** 10.1038/srep26258

**Published:** 2016-05-17

**Authors:** Hu Wang, Zhao-Hui Zhang, Zheng-Yang Hu, Fu-Chi Wang, Sheng-Lin Li, Elena Korznikov, Xiu-Chen Zhao, Ying Liu, Zhen-Feng Liu, Zhe Kang

**Affiliations:** 1 School of Materials Science and Engineering, Beijing Institute of Technology, Beijing 100081, PR China; 2 National Key Laboratory of Science and Technology on Materials under Shock and Impact, Beijing 100081, PR China; 3 Institute for Problems of Metals Superplasticity, Russian Academy of Sciences, ul. Khalturina 39, Ufa 450001, Russia; 4 R&D Center for New Material and Processing System Division, Sumitomo Coal Mining Company, Ltd., Tokyo 1148513, Japan

## Abstract

In this study, a novel multi-walled carbon nanotubes reinforced nanocrystalline copper matrix composite with super high strength and moderate plasticity was synthesized. We successfully overcome the agglomeration problem of the carbon nanotubes and the grain growth problem of the nanocrystalline copper matrix by combined use of the electroless deposition and spark plasma sintering methods. The yield strength of the composite reach up to 692 MPa, which is increased by 2 and 5 times comparing with those of the nanocrystalline and coarse copper, respectively. Simultaneously, the plasticity of the composite was also significantly increased in contrast with that of the nanocrystalline copper. The increase of the density of the carbon nanotubes after coating, the isolation effect caused by the copper coating, and the improvement of the compatibility between the reinforcements and matrix as well as the effective control of the grain growth of the copper matrix all contribute to improving the mechanical properties of the composite. In addition, a new strengthening mechanism, i.e., the series-connection effect of the nanocrystalline copper grains introduced by carbon nanotubes, is proposed to further explain the mechanical behavior of the nanocomposite.

Copper is one of the most important engineering materials for thermal and electronic applications due to its higher electrical and thermal conductivities as well as the lower coefficient of thermal expansion (CTE)^1^. However, poor mechanical performances of pure copper limit the proliferation of the metal into other fields. Although the copper matrix composite (CMC) reinforced with ceramic fibers and particulates exhibit high specific strength and specific elastic modulus over their monolithic metal, the electrical and thermal properties of the composites are usually decreased^2^,^3^. Comparing with ceramic reinforcements, conducting carbon nanotubes (CNTs) are competitive reinforcing materials due to their high thermal conductivity, low CTE, high damping capacity and good self-lubricant property^4^,^5^. In addition, CNTs also have an outstanding reinforcement effect on copper matrix^6^. Moreover, the mechanical properties of the composites will be further increased if the nanocrystalline (NC) copper is used as the metal matrix^7^. Therefore, NC copper matrix composite (NCCMC) reinforced with CNTs are considered attractive to meet the increasing demands for high performance materials. This study exhibits a novel multi-walled CNTs (MWCNTs) reinforced NC copper matrix composite (MWCNTs/NCCMC) with super high strength and moderate plasticity.

As a novel and rapid powder consolidation process, the spark plasma sintering (SPS) technique enables metal matrix composites with good mechanical properties to be fabricated by providing much more rapid heating and cooling rates, a short holding time and a high pressure at a relatively low sintering temperature^8^. These characteristics effectively prohibit the grain growth of the NC copper matrix and simultaneously prevent the chemical reaction between MWCNTs and the Cu matrix during the sintering process. Kim, K. T. *et al*.^9^ have confirmed that the hardness and sliding wear resistance of CNT/Cu nanocomposite were enhanced by two and three times, respectively, compared to those of Cu matrix. The electrical and mechanical properties of CNT/Cu nanocomposites fabricated by SPS were discussed by Hong, S. H. and co-worker^10^,^11^. The results indicate that the homogeneously distribution of CNTs in matrix is the key issues to enhance the physical and mechanical properties of CNT/Cu nanocomposites.

Although some progress has been achieved in CNTs reinforced CMC (CNTs/CMC)^6^,^11^ , to the best of our knowledge, there is no report on the synthesis and properties research on CNTs/NCCMC^12^. The major challenge in the fabrication of CNTs/NCCMC is how to homogeneously disperse the CNTs into the NC Cu matrix. It is well known that the typical characteristic of CNTs in the metal matrix is the agglomeration effect due to the large density and physical properties difference between the matrix and the reinforcements^12^,^13^. Moreover, the agglomeration effect of CNTs will be aggravated when the NC metal matrix is used because of the nature of nanoparticles. Such an agglomerated state is highly undesirable. High energy ball milling is usually used to mix CNTs and metal powders to obtain a homogeneous nanomixture^14^. However, on the one hand the structures of the CNTs are easy to be damaged and on the other hand some impurities may be introduced into the mixtures due to the long time ball-milling process^15^. The both cases will surely lead to a significant decline in the comprehensive performance of the composites. Kim, K. T. *et al*.^16^ have corroborated that the enhanced yield strength of CNTs/CMCs fabricated by the high energy ball milling and cold rolling process mainly stemmed from the homogenous distribution of the reinforcements. However, there still exist a few agglomerated CNTs in the nanocomposites, which were demonstrated by the two-scale micromechanical model developed by Barai, P. *et al*.^17^.

Our strategy for developing a novel fabrication process for MWCNTs/NCCMC basically involves fabricating Cu coated MWCNTs, synthesizing Cu nanopowders, mixing Cu coated MWCNTs with copper nanopowders and fabricating MWCNTs/NCCMC. The first key process is to produce Cu coated MWCNTs powders through electroless deposition (ED) method. In this process, the MWCNTs were firstly processed to make the functional groups and the active sites generate on the surface of the MWCNTs, and then the ED method was adopted to achieve a uniform nanoscale copper coating on the surface of the MWCNTs. The nano copper coating can not only make the MWCNTs homogeneously disperse in nano Cu particles but also provide a high interface bonding strength (IBS) between the MWCNTs and Cu matrix^18^. The second key process is to rapidly consolidate the mixture powders at a relatively low temperature through SPS method. Rapid heating and cooling rate used in SPS process can effectively prohibit the grain growth of the NC copper matrix.

## Results

**Figure 1:**
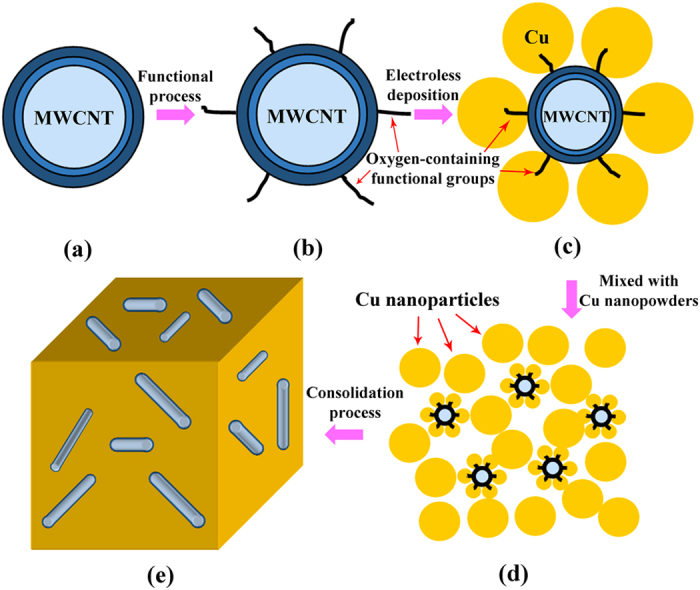
Schematics depicting the strategies and procedures for preparing the MWCNTs/NCCMC. (**a**) Purified MWCNTs. (**b**) Functionalized MWCNTs. (**c**) Cu coated MWCNTs. (**d**) MWCNTs/Cu nanocomposite powders. (**e**) MWCNTs/NCCMC with homogeneously distributed MWCNTs in the NC Cu matrix.

Figure 1 illustrates the steps of fabricating MWCNTs/NCCMC. First, the pristine MWCNTs were purified using hydrochloric acid to remove impurities (Fig. 1a). Second, the purified MWCNTs were functionalized. This process includes oxidation, sensitization and activation of the MWCNTs (Fig. 1b). The functionalized MWCNTs provide enhanced hydrophilicity to the surface of the MWCNTs, contributing to improving the dispersity of the MWCNTs in the aqueous solvents^19^. Third, the MWCNTs were coated with a layer of nano copper using ED method (Fig. 1c). Next, the MWCNTs/Cu nanocomposite powders were prepared by mixing Cu coated MWCNTs with Cu nanopowders in an anhydrous ethanol (Fig. 1d). Finally, the nanocomposite powders were sintered and densified by SPS process (Fig. 1e). The volume content of the MWCNTs in the composite is equal to 3%. For comparative study, NC Cu and coarse-grained (CG) Cu without any addition of MWCNTs were also prepared through SPS process under the same sintering conditions using the nano Cu powders and commercial Cu powders (99.9%, 200 mush), respectively.

**Figure 2:**
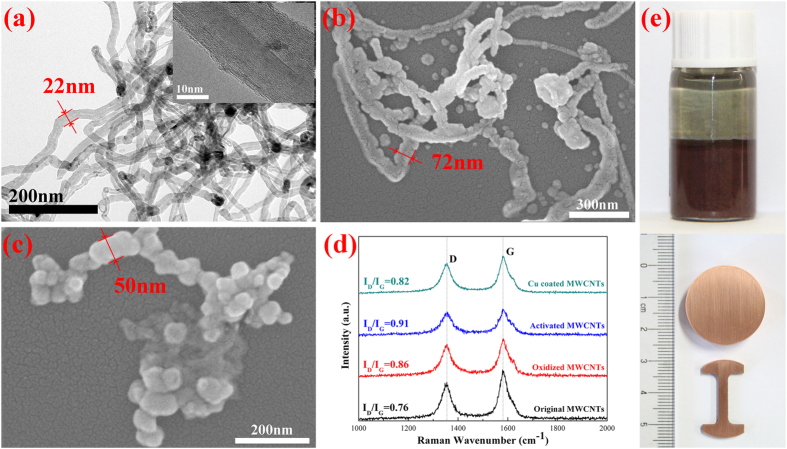
Characteristic of MWCNTs, copper nanopowders and MWCNTs/NCCMC nanocomposites. (**a**) TEM image of the pristine MWCNTs and the inset shows the HRTEM image of the longitudinal section of a MWCNT. (**b**) SEM micrographs of Cu coated MWCNTs. (**c**) SEM micrographs of Cu nanopowders. (**d**) Raman spectra of the MWCNTs obtained at different treatment stages. (**e**) MWCNTs/Cu nanocomposite powders and the consolidated MWCNTs/NCCMC.

Figure 2a shows the transmission electron microscope (TEM) image of the pristine MWCNTs and the inset shows the high-resolution TEM (HRTEM) image of the longitudinal section of a MWCNT. The image indicates that the average length, inner diameter and outer diameter of the MWCNTs are about 6 μm, 6 nm and 22 nm, respectively. Figure 2b shows that a uniform copper layer was coated on the surface of MWCNT and the diameter of the copper coated MWCNTs is about 72 nm (the deposition time is 15 min, Supplementary Figure 1 shows the SEM image of a MWCNT after 10 min deposition, indicating the mechanism of the ED method). Then it can be calculated that the average thickness of the copper coating is about 25 nm. The increase of the density of the MWCNTs after coating and the improvement of the compatibility between the MWCNTs and the copper nanoparticles as well as the isolation effect introduced by the copper coating is very beneficial to homogeneously dispersing MWCNTs in the Cu nanoparticles. Figure 2c presents the SEM image of the Cu nanoparticles, indicating that the average grain size of Cu nanoparticles is about 50 nm. Figure 2d shows the Raman spectra of the MWCNTs obtained at different treatment stages. The image reveals that the Raman spectra of the MWCNTs have two sharp peaks at 1355 cm^−1^ and 1581 cm^−1^ that are related to D band and G band. In addition, it can be observed that the I_D_/I_G_ ratio increased from 0.76 for original MWCNTs to 0.86 for oxidized MWCNTs, indicating that the oxygen-containing functional groups were generated on the surface of MWCNTs. However, the value of I_D_/I_G_ of the Cu coated MWCNTs powders were markedly lower (i.e., 0.82). The reason lies in that the deposition process removed most of the functional groups and partially recovered the nature of MWCNTs^20^. Figure 2e shows the MWCNTs/Cu nanocomposite powders and the consolidated MWCNTs/NCCMC.

**Figure 3:**
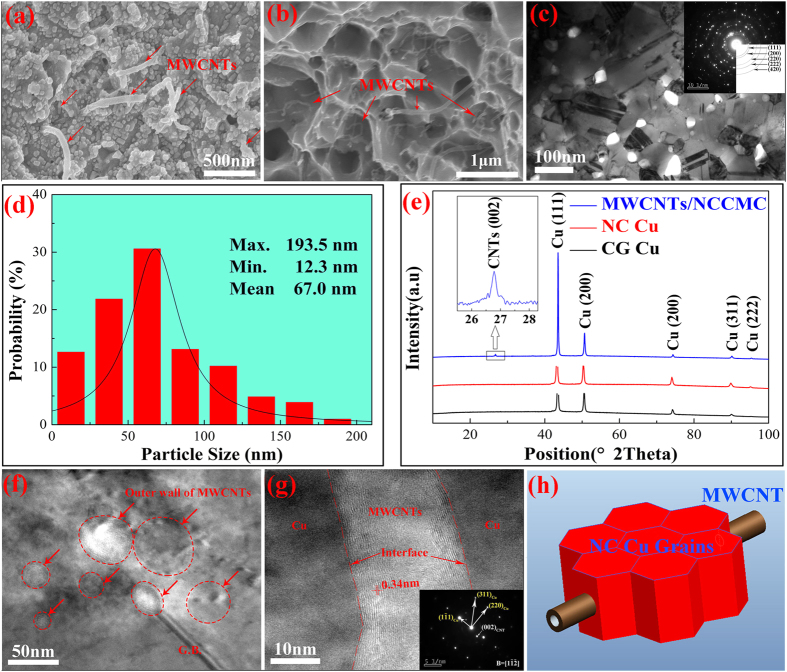
Characterization of MWCNTs/NCCMC nanocomposites. (**a**) SEM image of the MWCNTs/NCCMC after etching. (**b**) Fracture surface of the MWCNTs/NCCMC. (**c**) TEM image and the corresponding SAED patterns of the MWCNTs/NCCMC. (**d**) Histograms of the particle size distribution for the MWCNTs/NCCMC. (**e**) XRD patterns of the sintered compacts. (**f**) TEM image showing the cross sections of some non-agglomerated MWCNTs in the NC Cu matrix. (**g**) TEM image showing the longitudinal section of a MWCNT in the NC Cu matrix, the inset shows the SAED patterns of the corresponding analyzed area. (**h**) A schematic displaying the series-connection effect.

Figure 3a,b show the SEM image of the etched surface and the fracture surface of the MWCNTs/NCCMC, respectively, revealing that the MWCNTs were homogeneously distributed in the NC Cu matrix. Figure 3b also suggests that both the tensile fracture and pull out of the MWCNTs occurred on the fracture surface of the MWCNTs/NCCMC, indicating that not only the yield strength but also the plasticity of the composites should be increased in comparison with those of the NC Cu matrix. Figure 3c,d show a size analysis on the low-magnification TEM images of the MWCNTs/NCCMC, suggesting that the average size of the NC Cu matrix is about 67.0 nm. The selected area electron diffraction (SAED) pattern shown in Fig. 3c exhibits several bright rings, revealing that the nanocrystals were distributed at random. Figure 3e shows the XRD patterns of the sintered compacts. From the spectra, we can see that only the diffraction peaks of Cu and the MWCNTs are shown, and the prominent reflection in the diffraction profile at 26.6° (indexed as CNTs (002)) corresponds to the interplanar spacing of 0.34 nm of the CNTs.

Figure 3f shows the TEM image of the cross sections of some well-distributed MWCNTs in the NC Cu matrix. It can be observed that the MWCNTs with different diameters are located both at the Cu grain boundaries (GB) and in the interior of the Cu grains. The HRTEM image shown in Fig. 3f also reveals that a MWCNT is located inside a NC Cu grain. Considering the length and diameter of the MWCNTs and the grain size of copper matrix, we conclude that a single MWCNT may penetrate through some adjacent NC Cu grains and connect them together in the preparation procedure of the composites. This phenomenon is just like threading a few tomatoes on a steel wire. As a result, the IBS between the NC Cu grains were effectively enhanced and thus the mechanical properties of the nanocomposites were increased correspondingly^21^. We define the strengthening mechanism as “series-connection effect” and Fig. 3h displays this effect. In addition, Fig. 3g shows that no physical gaps exist at the interface between the MWCNTs and Cu matrix, also indicating that a high IBS has been achieved in the composites.

**Figure 4:**
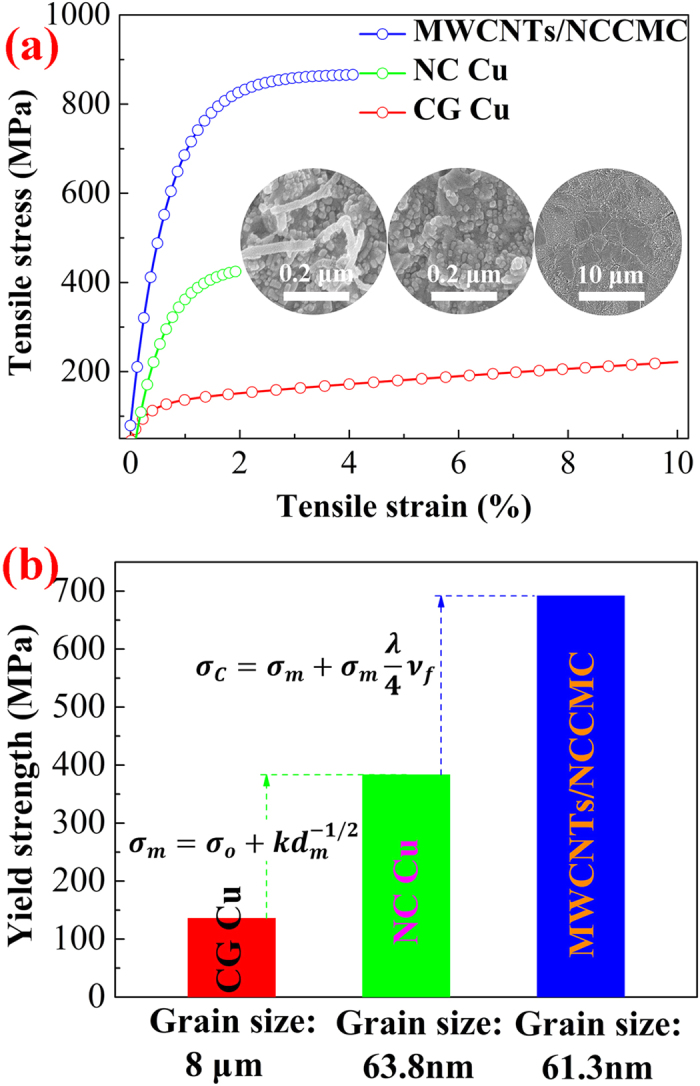
Mechanical properties of MWCNTs/NCCMC nanocomposites. (**a**) True tensile stress-strain curves of the MWCNTs/NCCMC, NC Cu and CG Cu, the inset shows the corresponding SEM images of these samples. (**b**) A comparison of the yield strength of the MWCNTs/NCCMC, NC Cu and CG Cu.

Figure 4a,b show the mechanical properties of the MWCNTs/NCCMC, NC Cu and CG Cu. The CG Cu and NC Cu have the average grain sizes of 8 μm and 70 nm, respectively (Table 1, obtained from Supplementary Figures 2 and 3). The yield strength of the NC Cu is about 383 MPa, which was about three times of that of the CG Cu (136 MPa). And the tensile strength of the NC Cu is also increased by 75% in comparison with that of the CG Cu. The results can obviously be attributed to the grain refinement effect (Fig. 4b)^22^. However, it is worth noting that the yield and tensile strength of the MWCNTs/NCCMC are 692 and 865 MPa, respectively, increasing by 80% and 99% in contrast with those of the NC Cu. Particularly, the yield strength of the composite is increased by 5 times comparing with that of the coarse copper. Such a high strengthening effect introduced by the MWCNTs is due to their homogeneous distributions in Cu matrix and the high IBS between the MWCNTs and Cu matrix.

## Discussion

The strengthening mechanism of the MWCNTs can be explained using the model developed by Nardone and Prewo^23^,^24^. According to this model, yield strength of the metal matrix composites can be calculated by Equation (1):
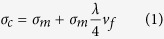
where *λ* and *v*_*f*_ are the aspect ratio and volume fraction of the MWCNTs, respectively, *σ*_*c*_ is the yield strength of the MWCNTs/NCCMC and *σ*_*m*_ is the yield strength of the Cu matrix. Considering that the saturation enhancement effect of MWCNTs, the critical aspect ratio (*λ*_*c*_) of the MWCNTs was proposed and regarded as the key factor to determine the yield strength of the nanocomposite. It can be defined as:
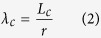
where *L*_*c*_ is the critical length of the MWCNTs, *r* is the average diameter of the MWCNTs (22 nm). The strengthening efficiency of the discontinuous fibers can be calculated based on the assumption that the matrix transmits the load to the fiber by means of the shear stresses that develop along the fiber–matrix interface^25^. A force balance allows:
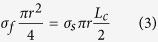
where *σ*_*f*_ is the tensile strength of the MWCNTs (about 39 GPa^26^) and *σ*_*s*_ is the shear strength of the copper matrix (≈*σ*_*m*_/2, *σ*_*m*_ = 383 MPa). Hence, *L*_*c*_ can be expressed as:
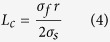


Combining Equation (2) and (4) yields:
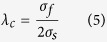


The calculated *λ*_*c*_ is equal to 101.7. Thus, according to Equation (1), the calculated *σ*_*c*_ is equal to 676 MPa (*v*_*f*_  = 0.03). The calculation result is close to the experimental ones. Thus, we can conclude that the increase of yield strength of the MWCNTs/NCCMC is mainly decided by the critical aspect ratio of the MWCNTs.

Furthermore, we can also explain the effect of the MWCNTs on strength of the composites by comparing their strengthening efficiency (*R*), which can be expressed as^11^:
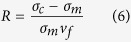


As shown in Table 2^11^,^18^,^25^,^27^, the strengthening efficiency of the MWCNTs in the MWCNTs/NCCMC prepared in the present study is about 26.8, which is much higher than those of the MWCNTs used in other nanocomposites. The result can be attributed to the homogeneous distribution of MWCNTs in metal matrix and the strong IBS between the reinforcements and the matrix^28^,^29^.

One interesting observation worth to be noted is that the elongation of the MWCNTs/NCCMC is about two times that of the NC Cu (Table 1). That is to say, the plasticity of the NC Cu is also increased by adding a small volume of MWCNTs into the nano metal matrix. In fact, poor plasticity is a common problem of nano metals or alloys fabricated by powder metallurgy method due to the low purity of the initial powders and the low density of the sintering compacts^30^,^31^,^32^. Pull out and bridge linking mechanism of the MWCNTs shown in Fig. 3b may be the main reasons for improving the plasticity of the MWCNTs/NCCMC^21^. In addition, a homogeneous distribution of the MWCNTs in metal matrix and the strong IBS between the reinforcements and the metal matrix may also contribute to improving the plasticity of the composite^33^,^34^.

In summary, this study demonstrates a MWCNTs reinforced NC copper matrix composite with super high strength and moderate plasticity. We successfully overcome the agglomeration problem of the MWCNTs and the grain growth problem of the NC Cu matrix by combined use of the ED and SPS methods. The synthesized MWCNTs/NCCMC is composed of nano-grained Cu matrix and well-distributed MWCNTs reinforcement. The increase of the density of the MWCNTs after coating and the isolation effect caused by the copper coating as well as the improvement of the compatibility between the reinforcements and metal matrix help to uniformly disperse the MWCNTs in copper matrix, which lead to the fabrication of MWCNTs/NCCMC with few agglomerated MWCNTs. Meanwhile, the low sintering temperature and the rapid heating and cooling rate employed in SPS process contribute to achieving the NC Cu matrix. All these factors help to increase the mechanical properties of the composites. In addition, the series-connection effect of the NC Cu grains introduced by the MWCNTs is also beneficial to improving the mechanical properties of the composites. As a result, the yield and tensile strength of the MWCNTs/NCCMC was greatly enhanced. Furthermore, the elongation of the MWCNTs/NCCMC was also increased in comparison with that of the NC Cu, due to the pull out and bridge linking mechanism of the MWCNTs.

## Methods

### Treatment of MWCNTs

The MWCNTs were synthesized using the catalytic chemical vapor deposition (CCVD) method. The so-obtained MWCNTs were purified with 200 mL HCl (35%) to remove the metallic catalyst. In order to deposit a thin and dense layer of copper on the surface of the MWCNTs, several pre-requisite steps were carried out before depositing. Firstly, the purified MWCNTs were acid-oxidized in a 3:1 mixture of H_2_SO_4_/HNO_3_ aqueous solutions with continuous high-speed stirring for 8 h in an ultrasonic bath at 50 °C. Subsequently, the oxidized MCWNTs were filtered with deionized water until the PH value reached 7.0 and then they were dried in vacuum at 50 °C. Secondly, the oxidized MWCNTs were sensitized using SnCl_2_/HCl solution with continuous stirring for 30 min in an ultrasonic bath. Then the sensitized MWCNTs were activated by ultrasonication method in PdCl_2_/HCl solution with stirring for 30 min.

### Fabrication of Cu coated MWCNTs and Cu nanopowders

The ED method was used to deposit a uniform layer of copper on the surface of the activated MWCNTs. Table 1 (see Supplementary information) shows the chemical composition of the bath used in the ED process. The deposition time was 15 min. In addition, the hydrazine hydrate chemical reduction method was adopted to fabricate Cu nanopowders. In this procedure, CuSO_4_·5H_2_O was used as the raw material. Hydrazine hydrate, ethylenediaminetetraacetic acid (EDTA), and polyvinyl-pyrolidone (PVP) were used as the reducer, complex agent and protector, respectively. The reaction temperature was selected as 70 °C. After the reaction was completed, the powders were filtered, washed by alcohol and placed in alcohol.

### Fabrication of MWCNTs/Cu nanocomposite powders

Cu nanopowders and Cu coated MWCNTs were dispersed separately in the ethanol solutions for 30 min using ultrasonication. Subsequently, these suspensions were gently blended together. After stirring for 120 min, the mixtures were oven-dried in vacuum at 50 °C.

### Fabrication of MWCNTs/NCCMC

The SPS equipment (DR.SINTER SPS-3.20) with a pulse duration of 3.3 ms and a vacuum of 1 Pa was employed to consolidate the MWCNTs/NCCMC. Sintering temperature was measured by a thermocouple inserted in a hole located in the wall of a cylindrical cemented carbide die. 25 g of the MWCNTs/Cu nanocomposite powders were loaded into a carbide die with 25 mm inner diameter. The sintering temperature was selected as 500 °C and the heating rate was set as 100 °C/min. The holding compressive pressure and holding time were 300 MPa and 3 min, respectively.

### Characterization tests

The density of the sintered compacts was measured using Archimedes’ method. The microstructures of the specimens were observed by SEM (Hitachi S-4800) and TEM (JEOL JEM 2100F).The MWCNTs were characterized by confocal Raman spectroscopy (CRM 200) using a wavelength of 514.5 nm. Tensile tests were performed using a uniaxial servo hydraulic testing machine at a constant strain rate of 1.0 × 10^−3^ s^−1^.

## Additional Information

**How to cite this article**: Wang, H. *et al*. Synergistic strengthening effect of nanocrystalline copper reinforced with carbon nanotubes. *Sci. Rep.* **6**, 26258; doi: 10.1038/srep26258 (2016).

## Supplementary Material

Supplementary Information

## Figures and Tables

**Table 1:** Relative density, average grain size and mechanical properties of the CG Cu, NC Cu and MWCNTs/NCCMC.

**Sample**	**Relative density (%)**	**Grain size (nm)**	**Micro hardness (GPa)**	**Yield strength (MPa)**	**Tensile strength (MPa)**	**Elongation (%)**
CG Cu	99.9	8 × 10^3^	0.4	136	248	19.4
NC Cu	99.8	69.8	1.3	383	434	2.0
MWCNTs/NCCMC	98.9	67.0	1.7	692	865	4.2

**Table 2:** Strengthening efficiency of MWCNTs in different metal matrixes.

Reinforcement		Matrix			Nanocomposites	Strengthening efficiency of CNTs (*R*)
**Type**	**Vol. (%)**	**Type**	**Grain size (nm)**	**Yield strength (MPa)**	**Yield strength (MPa)**	
MWCNTs	3	A2024^25^	100	560	780	13.1
MWCNTs	7	Co^18^	310	970	1500	7.8
MWCNTs	3	Al^27^	65	360	507	13.6
MWCNTs	10	Cu^11^	—	150	455	20.3
MWCNTs	3	Cu	83	383	692	26.8
