# Supplementary material for: Synergistic strengthening effect of nanocrystalline copper reinforced with carbon nanotubes
Source: Sci Rep. 2016 May 17;6:26258. doi: 10.1038/srep26258 (PMC4869099; doi:10.1038/srep26258)
Supplement: Supplementary Information [file srep26258-s1.doc]

**Synergistic strengthening effect of nanocrystalline copper reinforced with carbon nanotubes**

Hu Wang^1^, Zhao-Hui Zhang^1,2,*^, Zheng-Yang Hu^1^, Fu-Chi Wang^1,2^, Sheng-Lin Li^1^, Elena Korznikov^3^, Xiu-Chen Zhao^1,2^, Ying Liu^1,2^, Zhen-Feng Liu^1^, Zhe Kang^4^

^1^ School of Materials Science and Engineering, Beijing Institute of Technology, Beijing 100081, PR China

^2^ National Key Laboratory of Science and Technology on Materials under Shock and Impact, Beijing 100081, PR China

^3^ Institute for Problems of Metals Superplasticity, Russian Academy of Sciences, ul. Khalturina 39, Ufa 450001, Russia

^4^ R&D Center for New Material and Processing System Division, Sumitomo Coal Mining Company, Ltd., Tokyo 1148513, Japan

^*^ Corresponding author: Z-H. Z. (E-mail: zhang@bit.edu.cn)

**Supplementary Information**


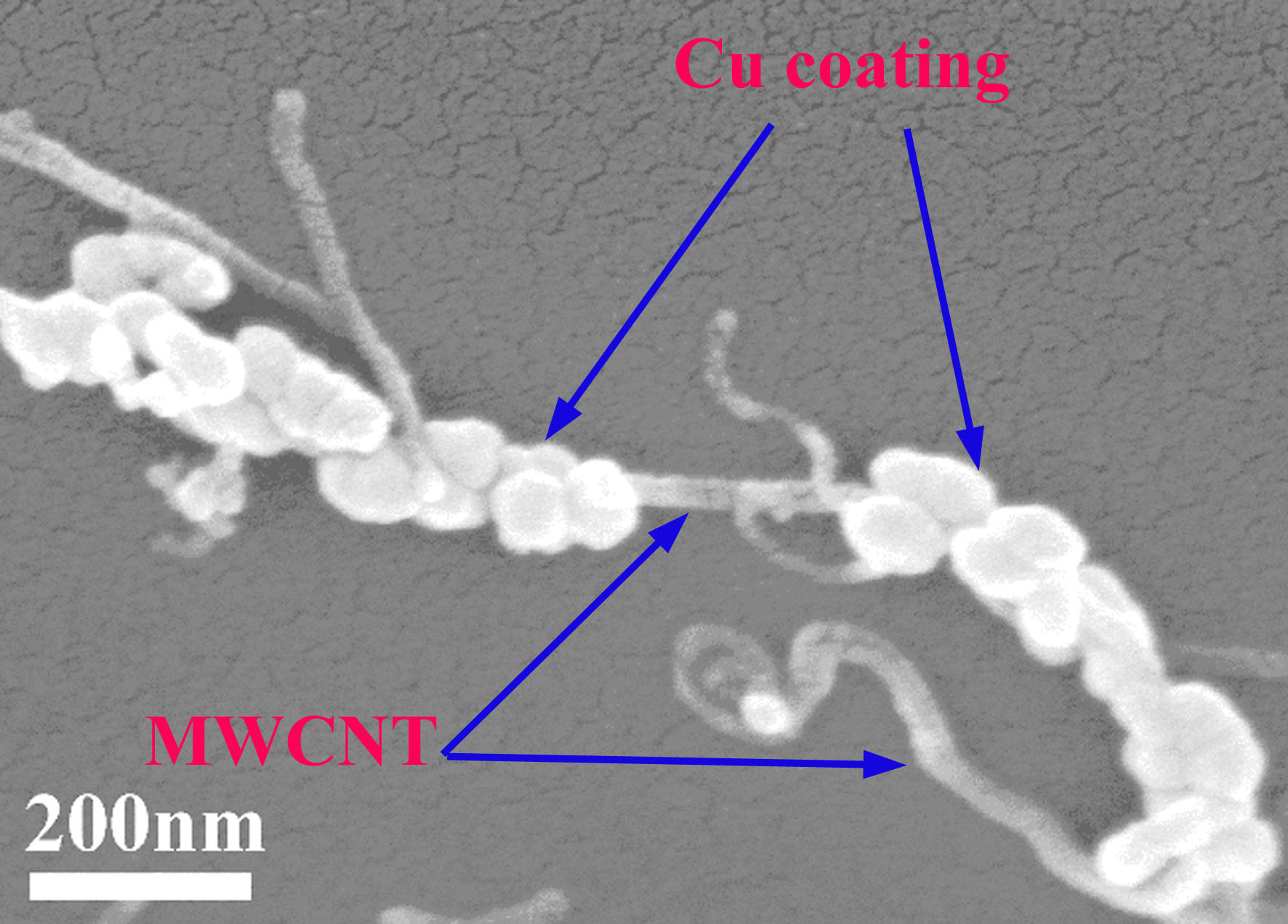


**Figure** **1** A typical SEM image of a MWCNT after 10 min deposition, indicating the mechanism of the ED method.


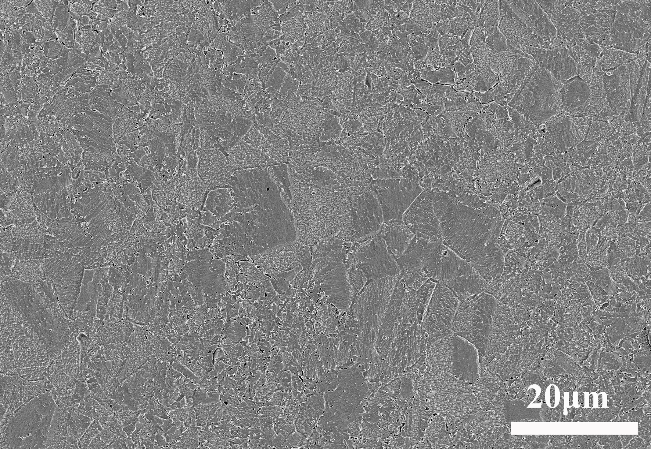


**Figure 2** Etched surface of the CG Cu after mechanical polishing and chemical etching.


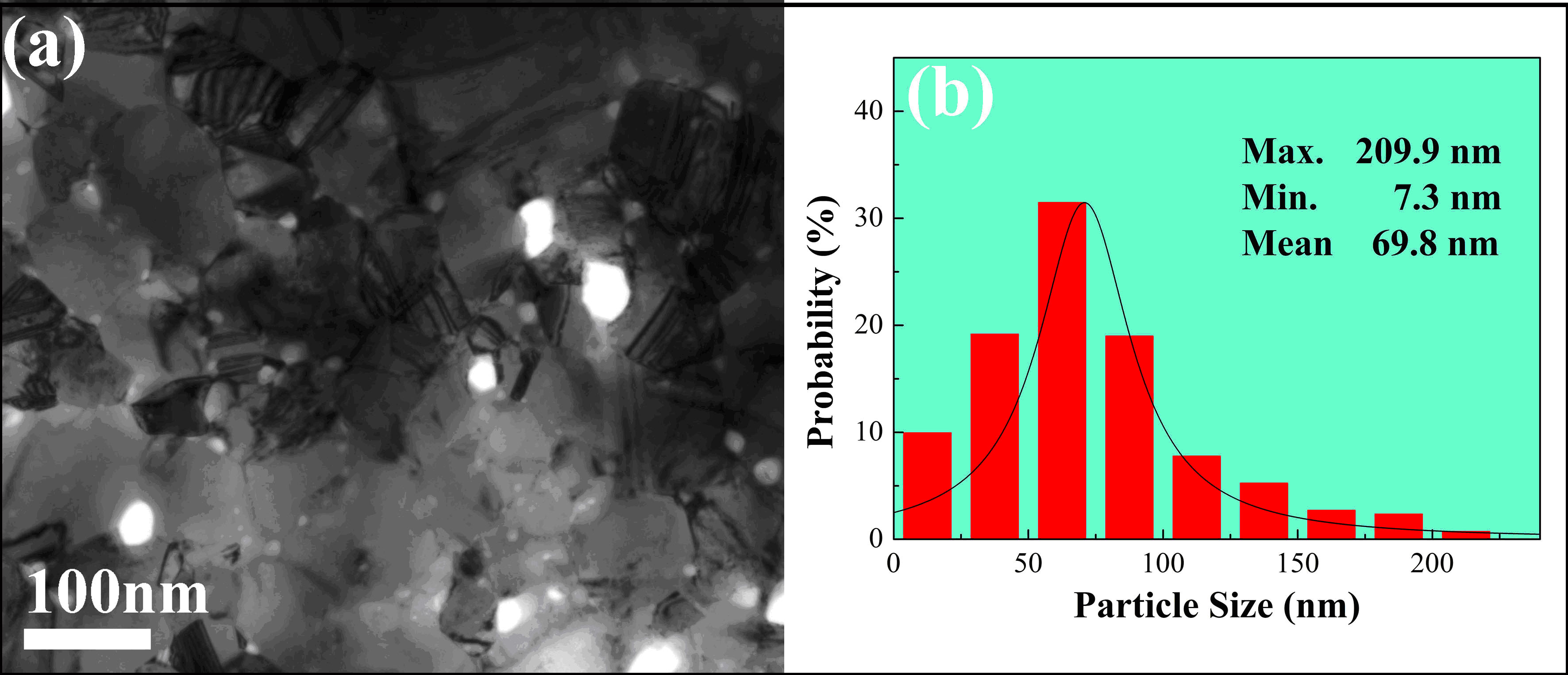


**Figure 3** (a) TEM image of the NC Cu. (b) Histograms of particle size distribution for the NC Cu.

**Table 1** Chemical composition of the electroless copper bath.

| Chemical composition | Quantity |
| --- | --- |
| CuSO_4_·5H_2_O (g/l) | 18 |
| Na_2_EDTA (g/l) | 40 |
| Formaldehyde (ml/l) | 20 |
| Activated MWCNTs (g/l) | 0.5 |
| pH | 11~12 |
| Temperature (°C) | 60 |
